# Data-sharing and re-analysis for main studies assessed by the European Medicines Agency—a cross-sectional study on European Public Assessment Reports

**DOI:** 10.1186/s12916-022-02377-2

**Published:** 2022-05-20

**Authors:** Maximilian Siebert, Jeanne Gaba, Alain Renault, Bruno Laviolle, Clara Locher, David Moher, Florian Naudet

**Affiliations:** 1 Univ Rennes, CHU Rennes, Inserm, CIC 1414 [(Centre d’Investigation Clinique de Rennes)], F-35000 Rennes, France; 2 Univ Rennes, CHU Rennes, Inserm, Irset (Institut de recherche en santé, environnement et travail) - UMR_S 1085, F-35000 Rennes, France; 3 Center for Journalology, Clinical Epidemiology Program, Ottawa Hospital Research Institute, Ottawa, Canada; 4 Clinical Investigation Center (Inserm 1414) and Adult Psychiatry Department, Rennes University Hospital, Rennes, France

**Keywords:** Reproducibility of results, Clinical trial, Drug approval

## Abstract

**Background:**

Transparency and reproducibility are expected to be normative practices in clinical trials used for decision-making on marketing authorisations for new medicines. This registered report introduces a cross-sectional study aiming to assess inferential reproducibility for main trials assessed by the European Medicines Agency.

**Methods:**

Two researchers independently identified all studies on new medicines, biosimilars and orphan medicines given approval by the European Commission between January 2017 and December 2019, categorised as ‘main studies’ in the European Public Assessment Reports (EPARs). Sixty-two of these studies were randomly sampled. One researcher retrieved the individual patient data (IPD) for these studies and prepared a dossier for each study, containing the IPD, the protocol and information on the conduct of the study. A second researcher who had no access to study reports used the dossier to run an independent re-analysis of each trial. All results of these re-analyses were reported in terms of each study’s conclusions, *p*-values, effect sizes and changes from the initial protocol. A team of two researchers not involved in the re-analysis compared results of the re-analyses with published results of the trial.

**Results:**

Two hundred ninety-two main studies in 173 EPARs were identified. Among the 62 studies randomly sampled, we received IPD for 10 trials. The median number of days between data request and data receipt was 253 [interquartile range 182–469]. For these ten trials, we identified 23 distinct primary outcomes for which the conclusions were reproduced in all re-analyses. Therefore, 10/62 trials (16% [95% confidence interval 8% to 28%]) were reproduced, as the 52 studies without available data were considered non-reproducible. There was no change from the original study protocol regarding the primary outcome in any of these ten studies. Spin was observed in the report of one study.

**Conclusions:**

Despite their results supporting decisions that affect millions of people’s health across the European Union, most main studies used in EPARs lack transparency and their results are not reproducible for external researchers. Re-analyses of the few trials with available data showed very good inferential reproducibility.

**Trial registration:**

https://osf.io/mcw3t/

**Supplementary Information:**

The online version contains supplementary material available at 10.1186/s12916-022-02377-2.

## Background

The influence of main studies (i.e. evidence used for drug marketing approval) as assessed by the European Medicines Agency (EMA) is paramount. These studies have a major impact on drug marketing authorisations and can change the practices of European medical practitioners and the care offered to millions of patients in the European Union. Because of the major financial conflicts of interest inherent in the evaluation of pharmaceuticals [1, 2], stakeholders are typically more confident when the results and conclusions of these studies can be verified. For a long time, however, transparency has been lacking and the individual patient data (IPD) and accompanying material (e.g. code, protocol, data analysis plan) to reproduce these analyses was unavailable. An empirical analysis suggests that only a small number of re-analyses of randomised controlled trials (RCTs) have been published to date; of these, only a minority were conducted by entirely independent authors [3]. Data-sharing enabling such re-analyses is being increasingly mandated in medicine.

And indeed, the EMA aimed to pioneer transparency in this field when, in November 2010, it decided to share all documentation received, in the wake of the first version of policy 0043 [4]. As part of its transparency policy, the EMA publishes European Public Assessment Reports (EPAR) after the European Commission’s decisions on the specific medicines. These reports include, among other documents, the results of main trials [5]. In October 2014, the EMA released its policy 0070 on “publication of clinical data for medicinal products for human use” [6]. The agency describes a two-step approach. From 1st of January 2015, clinical reports on medicines submitted for marketing authorisation have been published. A second step includes the publication of IPD. A date for the implementation of this step still needs to be fixed. However, as a result of Brexit and the relocation of the EMA to the Netherlands, further developments and renovation have been stopped for the moment [7, 8]. Efforts are therefore still needed to reach full transparency in the EMA.

On the other hand, umbrella groups of biopharmaceutical companies (i.e. Pharmaceutical Research and Manufacturers of America [PhRMA] and the European Federation of Pharmaceutical Industries and Associations [EFPIA]) endorsed a commitment “to enhancing public health through responsible sharing of clinical trial data” in a manner that is consistent with 3 main principles: (i) safeguarding the privacy of patients, (ii) respecting the integrity of national regulatory systems and (iii) maintaining incentives for investment in biomedical research [9]. Despite this commitment from 2013, an audit found that data availability was reached for only 9/61 (15%) clinical trials on medicines sponsored by the pharmaceutical industry and first published between 1 July 2015 and 31 December 2015 in the top 10 journals of general and internal medicine [10]. If such low rates of data-sharing were also to be observed for main trials, it would invalidate any efforts towards reproducibility for these important studies.

However, the environment for data-sharing is changing fast. And indeed, data-sharing platforms like ViVli, YODA project, or Clinical Study Data Request are more widely used. In the fall of 2019, these platforms gathered a large number of trials sponsored by the pharmaceutical industry. These three platforms included about 8000 RCTs in November 2019 [11]. Despite this available data, re-analyses are still sparse. Among the 88 published outputs we identified resulting from data-sharing on these platforms, only 3 were re-analyses: “Restoring Study 329” by Le Noury et al. which contradicted the initial publication, a trial that was already known to be misreported [12], a re-analysis of the TORCH trial suggesting an overestimation of the treatment effect in the original study [13] and the re-analysis of the “SMART-AF” trial which came to similar conclusions to the original study [14].

As part of a global research program on reproducibility in therapeutic research (ReiTheR, funded by the French National Research Agency), we designed the present cross-sectional study to assess inferential reproducibility (i.e. when IPD is available, whether qualitatively similar conclusions can be drawn from a re-analysis of the original trials) for main studies assessed by the EMA.

Our hypothesis is that for most trials (> 95%) for which we obtain the data, the results observed on the primary outcome would be fully reproducible. However, although we planned 1 year for data collection, we are aware that after this time some data would still not be available and thus not be re-analysable. Nevertheless, the worst-case scenario for precision estimates is that 50% of the studies would be analysable and reproduced.

## Methods

This is a registered report: the research protocol was peer-reviewed by the journal before the actual research took place, and it received in-principle acceptance on December 20, 2019, and was registered on January 14, 2020, on the Open Science Framework [15].

Once accepted, the editors undertake to publish the completed study if the protocol is validated even if there are statistically negative findings (i.e. study hypothesis not verified). This approach is expected to reduce issues such as publication bias [16].

### Eligibility criteria

#### EPARs

We collected all EPARs on new authorised human medications, biosimilars and orphan medicines given a positive opinion by the Committee for Medicinal Products for Human Use (CHMP) between 1 January 2017 and 31 December 2019 and approved by the European Commission. EPARs concerning generics and hybrid medicine were excluded. Definitions concerning the different types of drugs can be found in the web appendix (Additional file 1: Table S1) [15]. The distinction between new biosimilars, new generics, new hybrid medicine, orphan medicines, or new medicines followed the CHMP Meeting Highlights [17].

#### Main studies

Pivotal trials are referred to as “main studies” in the different EPARs. Any main study was included, with no distinction in terms of study phase, study type, study design, or intervention.

If an indication for a drug had been refused and another indication authorised, the main study for the non-authorised indication was not considered.

Furthermore, studies with no primary outcome identified were not included and were listed as non-evaluable studies.

### Search strategy

#### Eligible main trials

Two reviewers (MS, JG) independently extracted all names of the new medicines, biosimilars and orphan medicines approved by the CHMP and entered the information on a standard data extraction form. Afterwards, a check was performed to verify that the CHMP opinion was adopted by the European Commission [18]. Next, the reviewers identified the corresponding eligible EPARs on the EMA website [19] and independently extracted all main studies reported in these EPARs. Disagreements were resolved by discussion between the two reviewers or after referral to a third reviewer (CL or FN) until a consensus was reached.

#### Sample size calculation

A random sample of 62 of these main studies was selected using R (rnorm function) [20]. This sample size ensured a precision of ± 12% to estimate our primary outcome (i.e. percentage of reproducible studies, see below for a definition) in the worst-case scenario for precision estimations (i.e. if the percentage of reproducible studies is 50%).

#### Main study document accessibility

For all randomly sampled studies, one reviewer (JG) searched for the EudraCT number and/or the Sponsor Protocol Number, and/or any other identification information in each EPAR and identified the official sponsor of the study. If this information was lacking, the same reviewer started a wildcard search using keywords (disease, drug) from the study in the European Union Clinical Trial Register [21]. If this was not successful, the reviewer went on the websites ClinicalTrials.gov [22], International Clinical Trials Registry Portal (ICTRP), World Health Organization [23] and the International Standard Randomised Controlled Trial Number (ISRCTN) allocated by BioMedCentral [24]. If information on sponsor and study number was still lacking, the reviewer contacted the EMA.

Once the sponsor and the study number were identified, the reviewer contacted the sponsor to collect all of the following *main study documents*: (i) IPD; (ii) data analysis plan; (iii) unpublished and/or published study protocols with any date-stamped amendments; (iv) all the following dates: date of the last visit of the last patient, date of database lock (if available) and date of study unblinding; and (v) unpublished and/or published (scientific article) study reports.

To this end, the reviewer sent a standardised email (Additional file 2: Letter 1), presenting the research project with a link to the registered protocol on the Open Science Framework [15]. In order to improve the return rate, up to 4 emails were sent, the original and 3 reminder emails (with a two-week interval between them).

When asked, we indicated that the data-sharing of raw data was welcome in the form of Study Data Tabulation Model (SDTM) which was created by the Clinical Data International Standard Consortium (CDISC) [25].

In some cases, it was sufficient to contact the sponsor by e-mail; in other cases, the sponsor asked us to retrieve the data on a data-sharing platform.

In parallel the same reviewer searched for these documents on the EMA portal [26] and by inspecting the published reports (if available) identified using *open trial* [27, 28]. This process is summarised in the web appendix (Additional file 3: Figure S1).

### Data extraction

The identification of main studies and the following trial characteristics were extracted from the EPARs on a standard data extraction form by two independent researchers (JG and FN). For each study, the following information was collected: patient characteristics (e.g. percentage of women, mean age of participants, paediatric indication), study methods (e.g. type of endpoint, description for each primary endpoint) and intervention characteristics (e.g. drug). An exhaustive list of the trial characteristics extracted can be found in the web appendix (Additional file 4: Table S2).

Concerning the re-analysis, a first reviewer (JG) collected the information and collated data for the re-analysis. More specifically, the reviewer prepared a dossier with the following information for each study: (i) the protocol; (ii) all amendments to the protocol (with their dates); (iii) all the following dates: date of the last visit of the last patient, date of database lock (if available) and date of study unblinding; and (iv) the IPD. If information was still lacking, the study authors were contacted.

### Strategy for re-analyses

If the IPD was not available 1 year after our initial request, we initially planned to consider the study as non-reproducible (primary outcome of our study). However, we allowed some flexibility deviations to this rule (in terms of delay) during the conduct of the study, since delays were in general longer than initially planned, including from the legal review on our side. We only considered studies as not reproducible when data was not shared entirely to reproduce the primary endpoint.

Based on the dossier prepared by the first reviewer, re-analyses of the primary outcome(s) of each study were performed by a second reviewer (MS) who had no access to study reports, journal publications, statistical analysis plan, or analytical code, in order to ensure that the analysis was as blind as possible to the primary analysis. In addition, this reviewer was instructed not to try to find these documents or the published report.

For single-blind studies or open-label studies, analyses were performed according to the first version of the protocol, because outcome switching has been documented. For double-blind studies, all re-analyses were based on the latest version of the protocol issued before database lock and unblinding. If this information was not available, the date of the last visit of the last patient was used as a proxy.

Although in therapeutic research, statistical analysis can be “routine”, in some cases the re-analyses involve difficult methodological choices. An independent senior statistician (AR) was available to discuss any difficult aspect or choice in the analysis plan before the re-analysis, so as to choose the most consensual analyses (e.g. intention-to-treat population for a superiority trial).

If insufficient information concerning the main analysis was provided in the protocol, the best practices for clinical research were used, following the International Council for Harmonisation of Technical Requirements for Pharmaceuticals for Human Use (ICH Guidelines) [29].

An analysis plan was developed for each study included and was recorded on the Open Science Framework. In the supplementary material, a table is provided with details of what was taken from the ICH guidelines in case of missing information (Additional file 5: Table S3).

Re-analyses entailed the following different steps: (i) identification of the primary outcome (and detection of outcome switching), (ii) definition of the study population, (iii) re-analysis of the primary outcome. Any change identified between the first version of the protocol and the version used for the re-analysis of the primary outcome was tracked and described.

### Procedure to assess reproducibility

All results of these analyses were reported in terms of each study’s (i) conclusion (positive or negative), (ii) *p*-value, (iii) effect size (and details about the outcome) and (iv) changes from the initial protocol regarding the primary outcome. Regarding point (i), a non-inferiority trial was considered positive when it showed non-inferiority.

These results were first compared with the results of the analyses reported in the EPARs and, if these were not available, with the study reports, and again if not available, with the publications. All results from all available documents were gathered (EPARs, study reports and publications) and were presented in the results section.

Because interpreting an RCT involves clinical expertise, and cannot be reduced to solely quantitative factors, an in-depth discussion between two researchers not involved in the re-analysis (JG and FN), based on both quantitative and qualitative (clinical judgement) factors, enabled a decision on whether the changes in results described quantitatively could materialise into a change in conclusions.

If these two reviewers judged that the conclusions were the same, the study results were considered as *reproduced*. If these two researchers judged that the conclusions were not the same, then the researcher in charge of the analysis (MS) was given the statistical analysis plan of the study and was asked to list the differences in terms of analysis. If he found a discrepancy between the study data analysis plan and his own analysis plan, then he corrected this discrepancy in his analysis (e.g. analysis population, use of covariates). Again, an in-depth discussion between two researchers not involved in the re-analysis (JG and FN) enabled a decision on whether the changes in results described quantitatively could materialise into a change in conclusions, and whether the differences in terms of analytical plan were understandable and acceptable. If these two researchers judged that the conclusions were the same, the study was considered as *reproduced with verification*.

If these two researchers judged that the conclusions were not the same or that the change in the analytical plan was neither justified nor desirable, a senior statistician performed his own re-analysis. Details on this step can be found in the protocol of the registered report [15]. This process is described in the web appendix (Additional file 6: Figure S2).

### Outcomes

The primary outcome is the proportion of studies where the conclusions were reproduced (yes/no; i.e. reproduced or reproduced with verification, as defined above). In case of a divergence for two or more co-primary outcomes in the same study (i.e. one analysis is reproduced and not the other(s)), the different co-primary outcomes were described independently but the whole study was considered as not reproduced. All reasons for classifying studies as non-reproducible or not reproduced were described qualitatively using a taxonomy we developed during the research process.

In addition, we described in what way the data-sharing required clarifications for which additional queries had to be presented to the authors to obtain the relevant information, to clarify labels or use, or both, and to reproduce the original analysis of the primary outcomes.

A catalogue of these queries was created, and we grouped similar clarifications for descriptive purposes to generate a list of some common challenges, and to help tackle these challenges pre-emptively in future published trials.

Concerning secondary outcomes, we described and compared the main outcomes, *p*-values and effect sizes in the re-analyses, and the analyses reported in the EPARs, the study reports and the publications, and we described discrepancies. In addition, for each paper, we assessed the presence of the following key reporting biases: selective reporting of the primary outcome and “spin” [30].

In case of outcome switching, meaning that a secondary outcome was considered as a primary outcome in the final analysis, both endpoints were to be re-analysed.

To analyse “spin” in the results observed for the primary outcome, we took the definition provided by Yavchitz et al. who described it as being “a specific way of reporting, intentional or not, to highlight that the beneficial effect of the experimental treatment in terms of efficacy or safety is greater than that shown by the results” [31].

The modalities of data-sharing were described by the following categories: the type of data-sharing, the time lapse for collecting the data, the reason for non-availability of data, the deidentification of data (i.e. 18 identifiers, as required by the Health Insurance Portability and Accountability Act) [32] and the type of the shared data (here we distinguish “computerized data” which is not formal or ordered, “cleaned data, categorized and ordered” and “analyzable data” meaning ready for analysis) [33].

### Data analysis

We performed a descriptive analysis of the characteristics of the main studies extracted included in the EPARs selected. This included counts, percentages and their associated 95% confidence intervals (CIs).

Effect estimates in the different studies were expressed as standardised mean differences (SMDs) and their associated 95% CIs. For binary outcomes, odds ratios and their 95% CIs were calculated and converted into the standardised mean difference [34].

To compare the results of our re-analyses with the original results, the following steps were implemented: (i) we compared the statistical significance in the form of the *p*-value. If different, the results were considered as not reproducible. If not different, (ii) we qualitatively compared effect sizes and their respective 95% CIs. In case of ± 0.10 points difference in point estimates (expressed as standardised mean differences), the difference was discussed with a clinician in order to assess its clinical significance.

All analyses were performed using the open source statistical software R (R Development Core Team) [20] and SAS software™ .

## Changes to the registered protocol

We set a 1-year deadline to obtain data. However, data demands were lengthy, and delays were in some cases produced from our side. Hence, study data that was sent after this date was included in the re-analysis process.

Furthermore, although we said we would only use R software for data analysis, SAS software was used for two studies because of its more potent approach in mixed model analyses.

For one study we were unable to calculate the odds ratio. Starting with the incidence rate ratio, we used Chinn conversion to receive obtain the SMD [35]. This approach is justified in cases where events are rare and the incidence rate ratio can be treated as an odds ratio.

Because of low data-sharing rates, one researcher checked (JG) whether data-sharing policies were posted on the companies’ websites. The findings were reconfirmed by a second researcher.

## Results

### Study selection

**Fig. 1:**
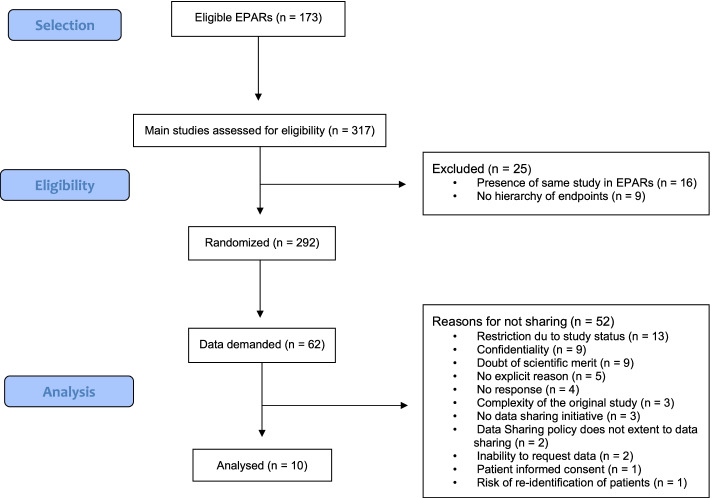
Flow chart for the selection and analysis process for main trials (EPAR: European Public Assessment Report)

The searches and consensus finished on 27 February 2020 and yielded 317 main studies identified in 173 EPARs. Of these, 25 were excluded (duplicates and studies with no primary endpoint) resulting in 292 individual studies. Of these, 62 were randomly selected (Fig. 1) and the respective data was requested from forty sponsors. All sponsors were contacted, and data was requested, either by mail or directly through a data-sharing platform. After exchanges with staff, for six datasets on Vivli, and for three on YODA, requests were issued.

### Data availability

Among the 62 studies, we received IPD for 10 trials (16% [CI95 8 to 28%]) from six sponsors [36–45]. For these studies, the median number of days before data became available was 253 [interquartile range (IQR) 182–469]. For these studies, all but one of the sponsors were big pharmaceutical companies and all but one of these companies had a data-sharing policy on their website. IPD for four studies was provided via data-sharing platforms (one was provided by one sponsor on Vivli and three by a single sponsor on YODA). Three studies were shared via a remote desktop monitored by the company in possession of the data. Another three data sets from three different sponsors were sent directly to us. All IPD received was analysable and deidentified.

**Fig. 2:**
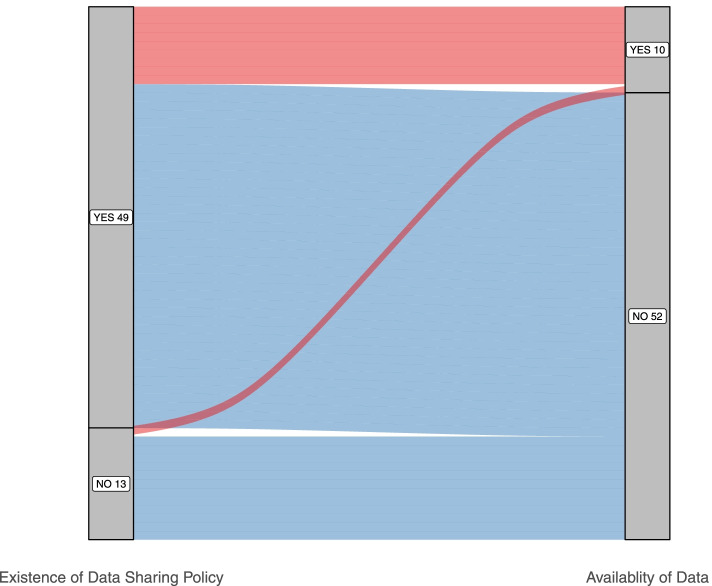
Outcome of data-sharing demands in relation to data-sharing policies

For the remaining 52 studies, reasons for unavailability were heterogeneous (Fig. 1). The most common reason was restriction due to the study status, i.e. extension studies were ongoing (13/52; 25%). Other reasons included confidentiality (9/52;17.3%) or lack of scientific merit as assessed by the companies’ procedures. The existence of possible privacy concerns was put forward for one study as a reason for not sharing data. Of the 52 studies where IPD was not shared, 40 (77%) belonged to companies that had a data-sharing policy (Fig. 2).

### Study characteristics

The characteristics of the ten studies with available IPD are presented in Table 1. The median sample size was 548 patients [IQR 278–778]. Three were single-arm studies, one was a two-arm study, four were three-arm and two were four-arm. Two involved a non-inferiority design, and for all ten studies, the primary publications, the study protocols and the study reports were retrieved.

**Table 1:** Summary of studies included for which data was received

Study acronym	Study	Study drug	Comparator drug	Sponsor	Design	Study duration (weeks)	Number of arms	Participants	Percentage of women	Mean age of participants (SD)
ENDURANCE-4	Asselah, 2018 [36]	Glecaprevir/pibrentasvir	NA	AbbVie Deutschland GmbH & Co. KG	Non-controlled cohort study	24	1	121	36.4	52.66 (11)
M41008−1002,	Mrowietz, 2017 [37]	Dimethyl fumarate	Placebo/dimethyl fumarate + ethyl hydrogen fumarate	Almirall, S.A.	Superiority and non-inferiority (head to head)	16	3	699	35.3	44.2 (14.5)
ERC 231	Archer, 2015 [38]	Dehydroepiandrosterone (DHEA)	Placebo	Endoceutics	Superiority (head to head)	12	3	255	100	58.5 (6)
Clarity	Giovannoni, 2010 [39]	Cladribine	Placebo	Merck Serono International S.A.	Superiority (head to head)	96	3	1326	67.6	38.6 (10)
NN7088-3885	Trakymiene, 2020 [40]	Turoctocog Alfa pegol	NA	Novo Nordisk A/S	Non-controlled cohort study	26	1	68	0	6 (3.3)
SUSTAIN 2	Ahren, 2017 [41]	Semaglutide	Sitagliptine	Novo Nordisk A/S	Superiority and non-inferiority (head to head)	56	4	1231	49.4	55.1 (10)
SUSTAIN 5	Rodbard, 2018 [42]	Semaglutide	Placebo	Novo Nordisk A/S	Superiority (head to head)	30	4	397	43.9	58.8 (10.1)
TRANSFORM-1	Fedgchin, 2019 [43]	Esketamine	Placebo	Janssen-Cilag International NV	Superiority (head to head)	4	3	346	70.3	46.3 (11.6)
SUSTAIN-1	Daly, 2019 [45]	Esketamine	Placebo	Janssen-Cilag International NV	Superiority (head to head)	16	2	705	64.8	46.1 (11.1)
SUSTAIN-2	Wajs, 2020 [44]	Esketamine	NA	Janssen-Cilag International NV	Non-controlled cohort study	52	1	802	62.6	52.2 (13.7)

### Reproducibility

**Fig. 3:**
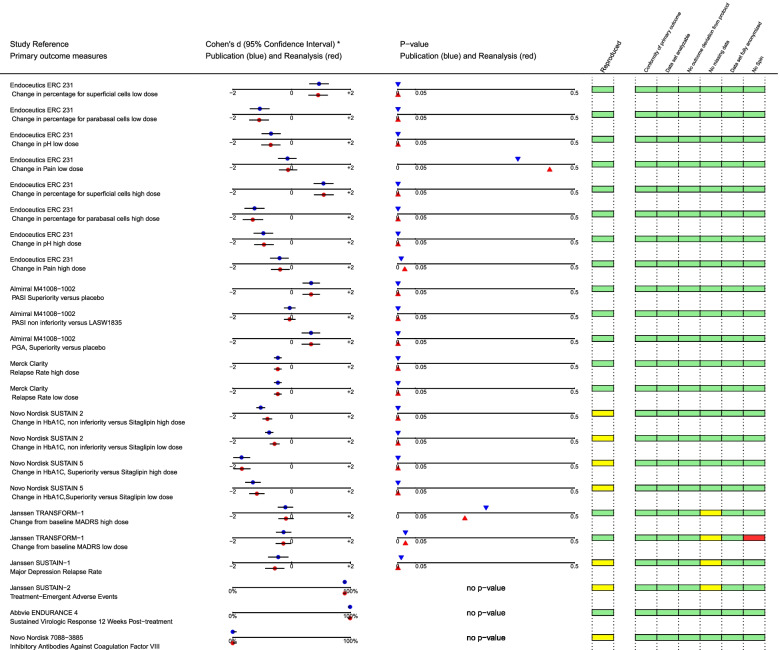
Outcome of study re-analyses in terms of effect size and *p*-values, including study details (* outcomes of three studies were expressed in percentages)

For the ten trials with available IPD, we identified 23 distinct outcomes eligible for re-analyses (relating to different comparisons and/or different primary endpoints). Detailed results of these re-analyses are presented in Fig. 3. Sixteen re-analyses (from six studies) were considered as reproduced; seven re-analyses (from five studies) were considered as reproduced with verification.

The 52 studies without available data were considered as not reproducible. Therefore, for our primary outcome, the conclusions of 10/62 trials (16% [CI95 8 to 28%]) were reproduced (i.e. reproduced (*n*= 5) or reproduced with verification (*n*= 5)).

We found no selective reporting of the studies’ primary outcomes and no change from the original study protocol for the primary outcome in any of these ten studies. Spin was observed in one study (see Table 2) [43].

**Table 2:** Identification of spin one of the selected studies

One study that examined the use of esketamine in treatment-resistant depression used a hierarchical testing approach: if the higher of two doses was not positive, according to the protocol the lower dose should not have been tested and reported. It was nevertheless tested and presented in the paper despite a negative result on the 84 mg dose: “… *Although esketamine 56 mg/ antidepressant could not be formally tested, the LS means difference was –4.1 [–7.67, –0.49] (nominal 2-sided P value=.027)*” and “*… Statistical significance was not achieved for the primary endpoint; nevertheless, the treatment effect (Montgomery-Asberg Depression Rating Scale) for both esketamine/antidepressant groups exceeded what has been considered clinically meaningful for approved antidepressants vs placebo [...] This study provides supportive evidence for the safety and efficacy of esketamine nasal spray as a new, rapid-acting antidepressant for patients with treatment- resistant depression.”*	

**Fig. 4:**
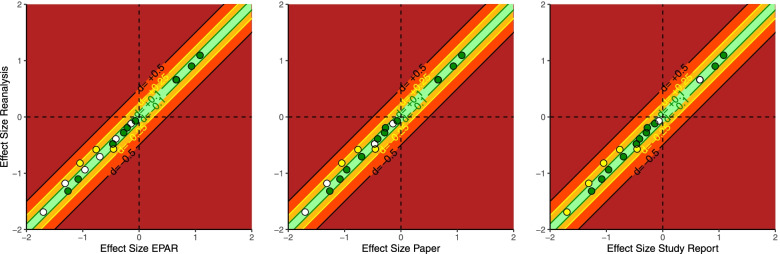
Original study effect size versus replication effect size in different types of reports (d: Cohen’s *d*; colour of the dots: green = outcome reproduced, yellow = outcome reproduced with verification, white = outcome not reported in this publication type; colour of the bars represent the range of differences in point estimates between re-analysis and result in publication type: light green = ± 0.10, orange = ± 0.25, light red = ± 0.50, dark red ≥ 0.50; *not including three outcomes that were expressed in percentages)

For 9/10 studies, the results reported in the EPAR, the study report, and the publication were identical (Fig. 4). In one study [43], small numerical differences were observed, since the statistical approach required by the EMA for the EPAR (ANCOVA) was different from the approach required by the FDA (mixed model with repeated measures) and reported in the study report and the paper. In some cases, comparisons were not indicated in the paper nor in the study report (as detailed on Fig. 4).

### List of challenges

#### Time required for data retrieval

Requesting and receiving the data was time-consuming. Interactions with sponsors were, on some occasions, lengthy, especially if several were involved on the same data-sharing platform. For example, on Vivli, we submitted a data request concerning six studies from three sponsors. The sponsors raised various questions. In one study (NCT00927498), the ownership of the data created confusion. The trial data was purchased by Pfizer. However, Vivli informed us that Pfizer was not in possession of the data and referred us back to the original Principal Investigator of the study who no longer had any rights over the data. After clarification via the platform, access was denied by Pfizer for reasons of insufficient scientific merit of our approach.

As part of the data acquisition process, the legal department of our unit had to confirm the data agreement and this step on our side was also lengthy. For two datasets, we exceeded our 1-year limit for data retrieval by 2 weeks. For three additional studies that were requested on YODA, 134 additional days were calculated. In this case, the data was not available at the time of our initial request (18/05/2020) but YODA contacted us on (04/03/2021) to indicate that the data was now on the platform and could be requested. After a request, we received this data on (14/10/2021). These studies were included in our analysis since these long time-lapses were considered as minor deviations from our initial protocol.

#### Incomplete datasets, metadata and further clarifications

Data dictionaries were available for 7/10 studies. In five studies, we had to contact the sponsor/platform to request additional data, as the data necessary to re-analyse the outcomes was missing (in three esketamine trials and in two semaglutide trials). We received additional data after 28 days in the case of the esketamine trials and were able to re-analyse the primary outcome. In the two three-arm studies from the same sponsor, comparing semaglutide with sitagliptin and placebo—non-inferiority on a primary outcome (change in HbA1c) and superiority on a “key secondary outcome” (bodyweight)—data concerning bodyweight was not available and was deleted as part of the anonymisation process. This study used a hierarchical testing approach and we initially planned to re-analyse the outcome relating to bodyweight which was distinct from the other secondary outcomes by being included in the hierarchical approach. Still, after contacting the sponsor who pointed out that it was not strictly speaking a primary outcome, we did not consider the analysis of bodyweight any further.

#### Data analysis

In 2/10 cases, while we reproduced the conclusions of the studies, we did not define the same analysis population with respectively 303 and 434 vs 297 and 433 patients analysed in the studies by Janssen SUSTAIN-1 and Merck Clarity. For the latter study, discrepancy for one patient was clarified with the sponsor. In this study, one patient was counted twice due to re-screening, and to the de-anonymisation of the data which made identification impossible. This information was included in the analysis data reviewer’s guide to which the authors had no access.

Among those two studies, the esketamine study published by Janssen had a very complex design involving randomised and non-randomised patients. The absence of a clear randomisation list and of a data dictionary made the re-analysis very challenging.

Two months after a request for clarification, we received the randomisation list. A close inspection of this list confirmed that we were able to correctly identify the 297 randomised patients and that we included by mistake in our analysis population six out of 600 non-randomised patients. Such discrepancy had no consequences on the conclusion of the re-analysis.

The results of the re-analysis exceeded the fixed threshold for the effect size of the primary endpoint of relapse of depressive symptoms (originally −0.45 vs −0.57 in the re-analysis) but this was considered a minor clinical difference and the study was considered as reproduced.

In studies using mixed models with repeated measures, we used SAS instead of R, reaching similar conclusions (suggested by the sponsor). However, small numerical inconsistencies were present. Again, for three outcomes in these two studies, the re-analysed effect size exceeded the prefixed threshold of 0.10 points in the effect size. However, the referees in charge (FN and JG) concluded that the differences of −1.05 vs −0.82, −0.76 vs −0.58 and −1.31 vs −1.18 for the change in HbA1c did not affect the conclusions of the study demonstrating large effect sizes in reducing HbA1c. The company in charge confirmed that due to anonymisation reproducing the exact same results would not be possible, even when providing the statistical code.

One study did not specify primary endpoints in its protocol but only objectives [44]. We double-checked the reasons for inclusion. Despite being a single-arm safety study, the trial was eligible since it was labelled as a main study in the EPAR and had primary endpoints described on ClinicalTrials.gov. The two researchers that were not involved in the study analysis decided to retain the first endpoint (treatment-emergent adverse events) over eleven primary outcomes listed on ClinicalTrials.gov for the analysis, as it was in line with the study objectives.

## Discussion

### Main results

Ten out of 62 main trials (16%) used by the EMA in its approval processes were reproduced. When IPD was available, all re-analyses largely reproduced the original results. These results are in line with an earlier survey of RCTs published by *PLOS Medicine* and *The BMJ* [46]. However, lack of IPD availability hampered our reproducibility effort for most of the trials, despite the fact that a large majority of sponsors had a data-sharing policy. It is clear that while pharmaceutical companies have signed on to the principles of data-sharing, they have not implemented this practice. Certain trials had extension phases, which, in the sponsors’ view, justified the non-data-sharing before study completion. Similar issues regarding the timing of the release of IPD have recently been described for COVID-19 vaccine trials [47].

These delays, rather like an embargo, could impact the possibility for independent researchers to perform timely re-analyses. Even for trials sharing IPD, times for requests and receipt of data were quite long. Another reason for non-availability of sharing was “lack of scientific merit” as assessed by the companies’ procedures. Interestingly, we intentionally adopted the registered report format for this paper, in order to pre-emptively address this potential concern: this publication process enabled a thorough and independent peer review of its “scientific merit” prior to data collection and analysis.

If the scientific merit of any data re-use is surely important when it comes to responsible sharing of IPD, it is however a subjective and arbitrary notion. Furthermore, there was no agreement on this point for our request, as some sponsors, including those with independent procedures (e.g. those sharing on YODA), agreed to share their data. It is likely that sponsors are less inclined to share their data for the purpose of a re-analysis. A survey of trialists suggested that willingness to share data could depend on the intended reuse of the data, with 97% of respondents willing to share data for a meta-analysis vs 73% for a re-analysis [48]. One additional explanation could be the fear of data misuse [49]. In addition, in the field of clinical trials, there is currently no systematic culture of reproducibility and independent re-analyses of clinical trials remain sparse in the published literature [3].

### Limitations

Caution is needed before generalising these results to other trials. Our results are focused on a very selective sample of trials, i.e. main studies submitted to the EMA. These studies (mostly from Europe) are larger than the average published RCT in the medical literature [50] and all were sponsored by the pharmaceutical industry. Implementation of data-sharing policies, although not optimal, is likely better than implementation by public funders [51]. In addition, we selected trials labelled as main studies (pivotal trials) in the EMA dossier and other studies could have been selected from the EPAR, i.e. the so-called supportive trials. Although less important, those supportive trials could have different characteristics from the main studies we included.

Low rates of data-sharing limited our ability to explore other inferential reproducibility issues in detail. In line with our registered protocol, 52 trials were categorised as non-reproducible because data-sharing was denied. In our definition, we considered that without the data, the results cannot be reproduced. However, the results of these missing studies could be reproducible if their individual patient data was available. The main result of our study is therefore that data-sharing is not implemented. In an ongoing complementary registered report that received in-principle acceptance in Royal Society Open Science [52], we have already received an agreement for 90% of 62 studies randomly selected on the main data-sharing platforms (Vivli, YODA and CSDR). These results will enable a triangulation of evidence on the reproducibility of therapeutic research.

Another limitation of our study is that it was restricted to primary endpoints. While primary endpoints are paramount in main trials, other endpoints (e.g. secondary endpoints and/or safety endpoints) could also be of interest to regulators. Furthermore, numerical differences, observed in some re-analyses, could be caused by the choices of the researcher in charge, and do not necessarily mean that the original estimates were wrong.

Finally, while we tried to ensure as far as possible that the re-analyst was blind to study results, some bias could have applied to the researcher in charge of re-analysing the data, as he was aware that the studies were part of authorised MAAs, which tend to be significantly “positive”, and indeed, all but one of the trials included were “positive”.

### Perspectives

Unlike the FDA, the EMA does not conduct independent re-analyses, making re-analyses by independent researchers even more important. Possibly, for these trials, the application of data-sharing policies should not rely only on the sponsor, and appropriate policies should be adopted by the regulatory authorities. While the EMA has demonstrated openness towards the idea of transparency with its implementation of 0043 policies and the first step in the even more progressive 0070 policy [53], more action is needed to ensure that data is effectively shared. Phase 2 of the EMA 0070 policy foresees the sharing of IPD, but there is no clear timeline yet. Our results support the urgent need to adopt, implement and monitor this policy.

In addition, efforts towards transparency and data-sharing could be incentivised. Success stories like the Good Pharma Score Card show that data-sharing rates rise when sponsors are made aware of its inaccessibility [54]. We have recently proposed the concept of registered drug approvals, an open science pathway for drug marketing authorisation which could incentivise data-sharing, among other open sciences practices [55].

## Conclusions

Data-sharing practices are rare for re-analyses of clinical trials for the authorisation of medication in Europe, even for sponsors with data-sharing policies. As a consequence, most main studies used in EPARs lack transparency and their results are not reproducible for external researchers, although their results support decisions that affect millions of people’s health across the European Union. Nonetheless, here re-analyses of the few trials with available data showed good inferential reproducibility. Our data provides a baseline for data-sharing implementation in these main studies. Europe strongly supports Open Science and transparency [56], it is therefore critical to develop interventions that increase data-sharing for these main studies, and to monitor improvements in the EMA data in the next few years.

## Supplementary Information

**Additional file 1: Table S1** Definitions of different types of medication on the EMA website.

**Additional file 2: Letter S1** Letter to the Sponsor.

**Additional file 3: Figure S1** Process of accessing main study documents.

**Additional file 4: Table S2** Study Characteristics extracted.

**Additional file 5: Table S3** Details of information extracted from the ICH guidelines in case of missing information.

**Additional file 6: Figure S2** Procedure for assessing reproducibility.

## Data Availability

Data and code that supports the findings of this study is made available on our link to the Open Science Framework (https://osf.io/mcw3t/). Furthermore, the pre-registered SAPs for the data analyses and a guide on how to demand IPD from the studies can be found under the link indicated above. Furthermore, interested researchers can contact the corresponding author via mail. IPD of the respective re-analysed studies cannot be shared directly but should be requested to the corresponding sponsor.

## Abbreviations

AdAM: Analysis data model
BMJ: The British Medical Journal
CDAS: Clinical Data Acquisition Standards Harmonisation
CDISC: Clinical Data International Standard Consortium
CHMP: Committee for Medicinal Products for Human Use
CI: Confidence interval
EFPIA: European Federation of Pharmaceutical Industries and Associations
EMA: European Medicines Agency
EPAR: European Public Assessment Report
EudraCT: European Union Drug Regulation Authorities Clinical Trials
ICH: International Council for Harmonisation of Technical Requirements for Pharmaceuticals for Human Use
ICTRP: International Clinical Trials Registry Portal
IPD: Individual patient data
ISRCTN: International Standard Randomised Controlled Trial Number
MAA: Marketing authorisation application
PhRMA: Pharmaceutical Research and Manufacturers of America
PLOS: Public Library of Science
RCT: Randomised controlled trial
ReiTheR: Reproducibility in therapeutic research
SDTM: Study Data Tabulation Model
SMD: Standardised mean difference

## References

[1] Als-Nielsen B, Chen W, Gluud C, Kjaergard LL (2003). Association of funding and conclusions in randomized drug trials: a reflection of treatment effect or adverse events?. JAMA..

[2] Djulbegovic B, Lacevic M, Cantor A, Fields KK, Bennett CL, Adams JR (2000). The uncertainty principle and industry-sponsored research. Lancet..

[3] Ebrahim S, Sohani ZN, Montoya L, Agarwal A, Thorlund K, Mills EJ (2014). Reanalyses of randomized clinical trial data. JAMA..

[4] European Medicines Agency. European Medicines Agency policy on access to documents. 2018. [cited 2019 July 05]. Available from: https://www.ema.europa.eu/en/documents/other/policy/0043-european-medicines-agency-policy-access-documents_en.pdf.

[5] European Medicines Agency. Guide to information on human medicines evaluated by EMA. 2017. Available from: https://www.ema.europa.eu/en/documents/other/guide-information-human-medicines-evaluated-european-medicines-agency-what-agency-publishes-when_en.pdf.

[6] European Medicines Agency. European Medicines Agency policy on publication of clinical data for medicinal products for human use. 2019. Available from: https://www.ema.europa.eu/en/documents/other/european-medicines-agency-policy-publication-clinical-data-medicinal-products-human-use_en.pdf.

[7] European Medicines Agency. Clinical data publication 2019 [cited 2019 June 24]. Available from: https://www.ema.europa.eu/en/human-regulatory/marketing-authorisation/clinical-data-publication.

[8] Doshi P (2018). EMA scales back transparency initiatives because of workload. BMJ..

[9] Pharmaceutical Research and Manufacturers of America. PhRMA Principles ForResponsible ClinicalTrial DataSharing 2013. Available from: http://phrma-docs.phrma.org/sites/default/files/pdf/PhRMAPrinciplesForResponsibleClinicalTrialDataSharing.pdf.

[10] Hopkins AM, Rowland A, Sorich MJ (2018). Data sharing from pharmaceutical industry sponsored clinical studies: audit of data availability. BMC Med.

[11] Ohmann C, Moher D, Siebert M, Motschall E, Naudet F (2019). Status, use and impact of sharing Individual Participant Data from clinical trials: a scoping review.

[12] Le Noury J, Nardo JM, Healy D, Jureidini J, Raven M, Tufanaru C (2015). Restoring Study 329: efficacy and harms of paroxetine and imipramine in treatment of major depression in adolescence. BMJ..

[13] Muff S, Puhan MA, Held L (2018). Bias away from the null due to miscounted outcomes? A case study on the TORCH trial. Stat Methods Med Res.

[14] Gay HC, Baldridge AS, Huffman MD (2017). Feasibility, process, and outcomes of cardiovascular clinical trial data sharing: A Reproduction Analysis of the SMART-AF Trial. JAMA Cardiol.

[15] Open Science Framework. Maximilian Siebert 2019. Available from: https://osf.io/mcw3t/.

[16] Hardwicke TE, Ioannidis JPA (2018). Mapping the universe of registered reports. Nat Hum Behav.

[17] European Medicines Agency. CHMP: Agendas, minutes and highlights 2019. [cited 2019 June 26]. Available from: https://www.ema.europa.eu/en/committees/chmp/chmp-agendas-minutes-highlights.

[18] European Commission. Public Health - Union Register of medicinal products 2019. [cited 2019 June 26]. Available from: http://ec.europa.eu/health/documents/community-register/html/index_en.htm.

[19] European Medicines Agency. Medicines 2019 [cited 2019 June 26]. Available from: https://www.ema.europa.eu/en/medicines.

[20] R Core Team. R (2018). A language and environment for statistical computing.

[21] European Medicines Agency. EU Clinical Trials Register 2019 June 25. [cited 2019 June 26]. Available from: https://www.clinicaltrialsregister.eu/ctr-search/search.

[22] U.S. National Library of Medicine. ClinicalTrials.gov 2019 [cited 2019 June 26]. Available from: https://clinicaltrials.gov/ct2/search.

[23] World Health Organization. International Clinical Trials Registry Platform Search Portal 2019 [cited 2019 June 26]. Available from: http://apps.who.int/trialsearch/.

[24] BioMedCentral. ISRCTN registry 2019 [cited 2019 June 26]. Available from: http://www.isrctn.com/.

[25] Hume S, Chow A, Evans J, Malfait F, Chason J, Wold JD (2018). CDISC SHARE, a global, cloud-based resource of machine-readable CDISC standards for clinical and translational research. AMIA Jt Summits Transl Sci Proc.

[26] European Medicines Agency. Clinical Data 2019 [cited 2019 June 26]. Available from: https://clinicaldata.ema.europa.eu/web/cdp/home.

[27] Goldacre B, Gray J (2016). OpenTrials: towards a collaborative open database of all available information on all clinical trials. Trials..

[28] Open Knowledge International, Data Lab. OpenTrials 2019 [cited 2019 June 26]. Available from: https://opentrials.net/.

[29] International Council for Harmonisation of Technical Requirements for Pharmaceuticals for Human Use. Welcome to the ICH official website 2019 [cited 2019 June 25]. Available from: https://www.ich.org/home.html.

[30] Chiu K, Grundy Q, Bero L (2017). 'Spin' in published biomedical literature: a methodological systematic review. PLoS Biol.

[31] Yavchitz A, Boutron I, Bafeta A, Marroun I, Charles P, Mantz J (2012). Misrepresentation of randomized controlled trials in press releases and news coverage: a cohort study. PLoS Med.

[32] U.S. Department of Health & Human Services. Guidance regarding methods for de-identification of protected health information in accordance with the Health Insurance Portability and Accountability Act (HIPAA) Privacy Rule 2012 [cited 2019 November 20]. Available from: https://www.hhs.gov/hipaa/for-professionals/privacy/special-topics/de-identification/index.html.

[33] Zarin DA, Tse T (2016). Sharing individual participant data (IPD) within the context of the trial reporting system (TRS). PLoS Med.

[34] Borenstein M, Hedges LV, Higgins JPT, Rothstein HR (2009). Introduction to meta-analysis.

[35] Chinn S (2000). A simple method for converting an odds ratio to effect size for use in meta-analysis. Stat Med.

[36] Asselah T, Kowdley KV, Zadeikis N, Wang S, Hassanein T, Horsmans Y (2018). Efficacy of glecaprevir/pibrentasvir for 8 or 12 weeks in patients with hepatitis C virus genotype 2, 4, 5, or 6 infection without cirrhosis. Clin Gastroenterol Hepatol.

[37] Mrowietz U, Szepietowski JC, Loewe R, van de Kerkhof P, Lamarca R, Ocker WG (2017). Efficacy and safety of LAS41008 (dimethyl fumarate) in adults with moderate-to-severe chronic plaque psoriasis: a randomized, double-blind, Fumaderm®- and placebo-controlled trial (BRIDGE). Br J Dermatol.

[38] Archer DF, Labrie F, Bouchard C, Portman DJ, Koltun W, Cusan L (2015). Treatment of pain at sexual activity (dyspareunia) with intravaginal dehydroepiandrosterone (prasterone). Menopause..

[39] Giovannoni G, Comi G, Cook S, Rammohan K, Rieckmann P, Sørensen PS (2010). A placebo-controlled trial of oral cladribine for relapsing multiple sclerosis. N Engl J Med.

[40] Šaulytė Trakymienė S, Economou M, Kenet G, Landorph A, Shen C, Kearney S (2020). Long-term safety and efficacy of N8-GP in previously treated pediatric patients with hemophilia A: final results from pathfinder5. J Thromb Haemost.

[41] Ahrén B, Masmiquel L, Kumar H, Sargin M, Karsbøl JD, Jacobsen SH (2017). Efficacy and safety of once-weekly semaglutide versus once-daily sitagliptin as an add-on to metformin, thiazolidinediones, or both, in patients with type 2 diabetes (SUSTAIN 2): a 56-week, double-blind, phase 3a, randomised trial. Lancet Diabetes Endocrinol.

[42] Rodbard HW, Lingvay I, Reed J, de la Rosa R, Rose L, Sugimoto D (2018). Semaglutide added to basal insulin in type 2 diabetes (SUSTAIN 5): a randomized, controlled trial. J Clin Endocrinol Metab.

[43] Fedgchin M, Trivedi M, Daly EJ, Melkote R, Lane R, Lim P (2019). Efficacy and safety of fixed-dose esketamine nasal spray combined with a new oral antidepressant in treatment-resistant depression: results of a randomized, double-blind, active-controlled study (TRANSFORM-1). Int J Neuropsychopharmacol.

[44] Wajs E, Aluisio L, Holder R, Daly EJ, Lane R, Lim P, et al. Esketamine nasal spray plus oral antidepressant in patients with treatment-resistant depression: assessment of long-term safety in a phase 3, open-label study (SUSTAIN-2). J Clin Psychiatry. 2020;81(3):19m12891. 10.4088/JCP.19m12891. 10.4088/JCP.19m12891 32316080

[45] Daly EJ, Trivedi MH, Janik A, Li H, Zhang Y, Li X (2019). Efficacy of esketamine nasal spray plus oral antidepressant treatment for relapse prevention in patients with treatment-resistant depression: a randomized clinical trial. JAMA Psychiatry.

[46] Naudet F, Sakarovitch C, Janiaud P, Cristea I, Fanelli D, Moher D (2018). Data sharing and reanalysis of randomized controlled trials in leading biomedical journals with a full data sharing policy: survey of studies published in The BMJ and PLOS Medicine. BMJ..

[47] Tanveer S, Rowhani-Farid A, Hong K, et al. Transparency of COVID-19 vaccine trials: decisions without data. BMJ Evid Based Med. 2021. 10.1136/bmjebm-2021-111735. 10.1136/bmjebm-2021-111735 34373256

[48] Tannenbaum S, Ross JS, Krumholz HM, Desai NR, Ritchie JD, Lehman R (2018). Early experiences with journal data sharing policies: a survey of published clinical trial investigators. Ann Intern Med.

[49] Rathi V, Dzara K, Gross CP, Hrynaszkiewicz I, Joffe S, Krumholz HM (2012). Sharing of clinical trial data among trialists: a cross sectional survey. BMJ Br Med J.

[50] Chan A-W, Altman DG (2005). Epidemiology and reporting of randomised trials published in PubMed journals. Lancet.

[51] Gaba JF, Siebert M, Dupuy A, Moher D, Naudet F (2020). Funders' data-sharing policies in therapeutic research: a survey of commercial and non-commercial funders. PLoS One.

[52] Gaba J, Siebert M, Renault A, Laviolle B, Locher C, Moher D, et al. Inferential reproducibility of therapeutic research: a cross-sectional study of randomized controlled trials available on major data-sharing platforms 2020. Available from: https://osf.io/dw8et/.

[53] Egilman AC, Kapczynski A, McCarthy ME, Luxkaranayagam AT, Morten CJ, Herder M (2021). Transparency of regulatory data across the European Medicines Agency, Health Canada, and US Food and Drug Administration. J Law Med Ethics.

[54] Bioethics International. Good Pharma Scorecard 2021. Available from: https://bioethicsinternational.org/good-pharma-scorecard/.

[55] Naudet F, Siebert M, Boussageon R, Cristea IA, Turner EH (2021). An open science pathway for drug marketing authorization—registered drug approval. PLoS Med.

[56] https://ec.europa.eu/info/research-and-innovation/strategy/strategy-2020-2024/our-digital-future/open-science_en

