# Supplementary material for: Data-sharing and re-analysis for main studies assessed by the European Medicines Agency—a cross-sectional study on European Public Assessment Reports
Source: BMC Med. 2022 May 20;20:177. doi: 10.1186/s12916-022-02377-2 (PMC9119701; doi:10.1186/s12916-022-02377-2)
Supplement: Supplementary file 1 — Additional file 1: Table S1 Definitions of different types of medication on the EMA website. [file 12916_2022_2377_MOESM1_ESM.docx]

**Table with definitions of different types of medicines on the EMA website (definitions were taken from the EMA website):**

| Generic medicine | A [generic medicine](https://www.ema.europa.eu/en/glossary/generic-medicine) contains the same [active substance](https://www.ema.europa.eu/en/glossary/active-substance)(s) as the reference medicine, and it is used at the same dose(s) to treat the same disease(s). However, a [generic medicine](https://www.ema.europa.eu/en/glossary/generic-medicine)'s inactive ingredients, name, appearance and packaging can be different.  [Generic medicines](https://www.ema.europa.eu/en/glossary/generic-medicine) are manufactured according to the same quality standards as all other medicines.  A company can only develop a [generic medicine](https://www.ema.europa.eu/en/glossary/generic-medicine) for marketing once the period of [data exclusivity](https://www.ema.europa.eu/en/glossary/data-exclusivity) on the reference medicine has expired. This is usually 10 years from the date of first authorization. (1) |
| --- | --- |
| Hybrid medicine | [Hybrid medicines](https://www.ema.europa.eu/en/glossary/hybrid-medicine) are medicines whose authorization depends partly on the results of tests on the reference medicine and partly on new data from [clinical trials](https://www.ema.europa.eu/en/glossary/clinical-trial).  This happens when a manufacturer develops a [generic medicine](https://www.ema.europa.eu/en/glossary/generic-medicine) that is based on a reference medicine, but has a different strength, a different [route of administration](https://www.ema.europa.eu/en/glossary/route-administration) or a slightly different [indication](https://www.ema.europa.eu/en/glossary/indication) from the reference medicine. (1) |
| Biosimilar | A biosimilar is a [biological medicine](https://www.ema.europa.eu/en/glossary/biological-medicine) highly similar to another [biological medicine](https://www.ema.europa.eu/en/glossary/biological-medicine) already approved in the EU (called 'reference medicine') in terms of structure, biological activity and [efficacy](https://www.ema.europa.eu/en/glossary/efficacy), safety and immunogenicity profile (the intrinsic ability of proteins and other [biological medicines](https://www.ema.europa.eu/en/glossary/biological-medicine) to cause an immune response).  The EU approved the first biosimilar in 2006.  A biosimilar is not regarded as a generic of a [biological medicine](https://www.ema.europa.eu/en/glossary/biological-medicine). This is mostly because the natural variability and more complex manufacturing of [biological medicines](https://www.ema.europa.eu/en/glossary/biological-medicine) do not allow an exact replication of the molecular micro-heterogeneity.  The EU has pioneered the regulation of [biosimilar medicines](https://www.ema.europa.eu/en/glossary/biosimilar-medicine) by establishing a solid framework for their approval and by shaping biosimilar development globally. (2) |
| Informed Consent Application | An informed consent application makes use of data from the dossier of a previously authorized medicine, with the [marketing authorization holder](https://www.ema.europa.eu/en/glossary/marketing-authorisation-holder) of that medicine giving consent for the use of their data in the application. (3) |
| Orphan Medicine | A medicine for the diagnosis, prevention or treatment of a life-threatening or chronically debilitating condition that is rare (affecting not more than five in 10,000 people in the European Union) or where the medicine is unlikely to generate sufficient profit to justify research and development costs. (4) |

1. European Medicines Agency. Generic and hybrid medicines 2019 [cited June 27 2019]. Available from: <https://www.ema.europa.eu/en/human-regulatory/marketing-authorisation/generic-hybrid-medicines>.

2. European Medicines Agency. Biosimilar medicines: Overview 2019 [cited 2019 June 25]. Available from: <https://www.ema.europa.eu/en/human-regulatory/overview/biosimilar-medicines-overview>.

3. European Medicines Agency. Informed consent application 2019 [cited 2019 June 27]. Available from: <https://www.ema.europa.eu/en/glossary/informed-consent-application>.

4. European Medicines Agency. Informed consent application 2019 [cited 2019 November 18].

Available from: <https://www.ema.europa.eu/en/about-us/about-website/glossary/name_az/O>
