# Supplementary material for: Data-sharing and re-analysis for main studies assessed by the European Medicines Agency—a cross-sectional study on European Public Assessment Reports
Source: BMC Med. 2022 May 20;20:177. doi: 10.1186/s12916-022-02377-2 (PMC9119701; doi:10.1186/s12916-022-02377-2)
Supplement: Supplementary file 2 — Additional file 2: Letter S1 Letter to the Sponsor. [file 12916_2022_2377_MOESM2_ESM.docx]

**Letter to the Sponsor:**

Dear XXX,

I am a PhD student at Rennes 1 University working in Rennes Clinical Research Investigation Center (CIC Inserm 1414) under the supervision of Florian Naudet (MD, PhD). My research team and I are interested in re-analyzing the primary outcomes of main studies submitted to the EMA.

Your study ‘XXXX’ reported in the EPAR “XXX” is one such trial and has been selected for re-analysis. Therefore, I would greatly appreciate if you could share the following documents from the trial with us: 1) IPD, 2) data analysis plan, 3) unpublished and/or published study protocol with any date-stamped amendments, 4) all the following dates: date of the last visit of the last patient, date of database lock (if available) and date of study unblinding, and 5) unpublished and/or published (scientific article) study reports.

Sharing in form of the Standard Tabulation Data Model (STDM) developed by the Clinical Data International Standard Consortium is welcome but not mandatory.

If you cannot share any of these data, please indicate the reason for not sharing.

If there are any questions do not hesitate to write to us or to consult our preregistered protocol under the following link: XXX.

Sincerely,

Jeanne Gaba

PhD Student,

Univ Rennes, CHU Rennes, Inserm, CIC 1414 (Centre d’Investigation Clinique de Rennes), 35000 Rennes, France
