# Supplementary figures and images for: Data-sharing and re-analysis for main studies assessed by the European Medicines Agency—a cross-sectional study on European Public Assessment Reports
Source: BMC Med. 2022 May 20;20:177. doi: 10.1186/s12916-022-02377-2 (PMC9119701; doi:10.1186/s12916-022-02377-2)

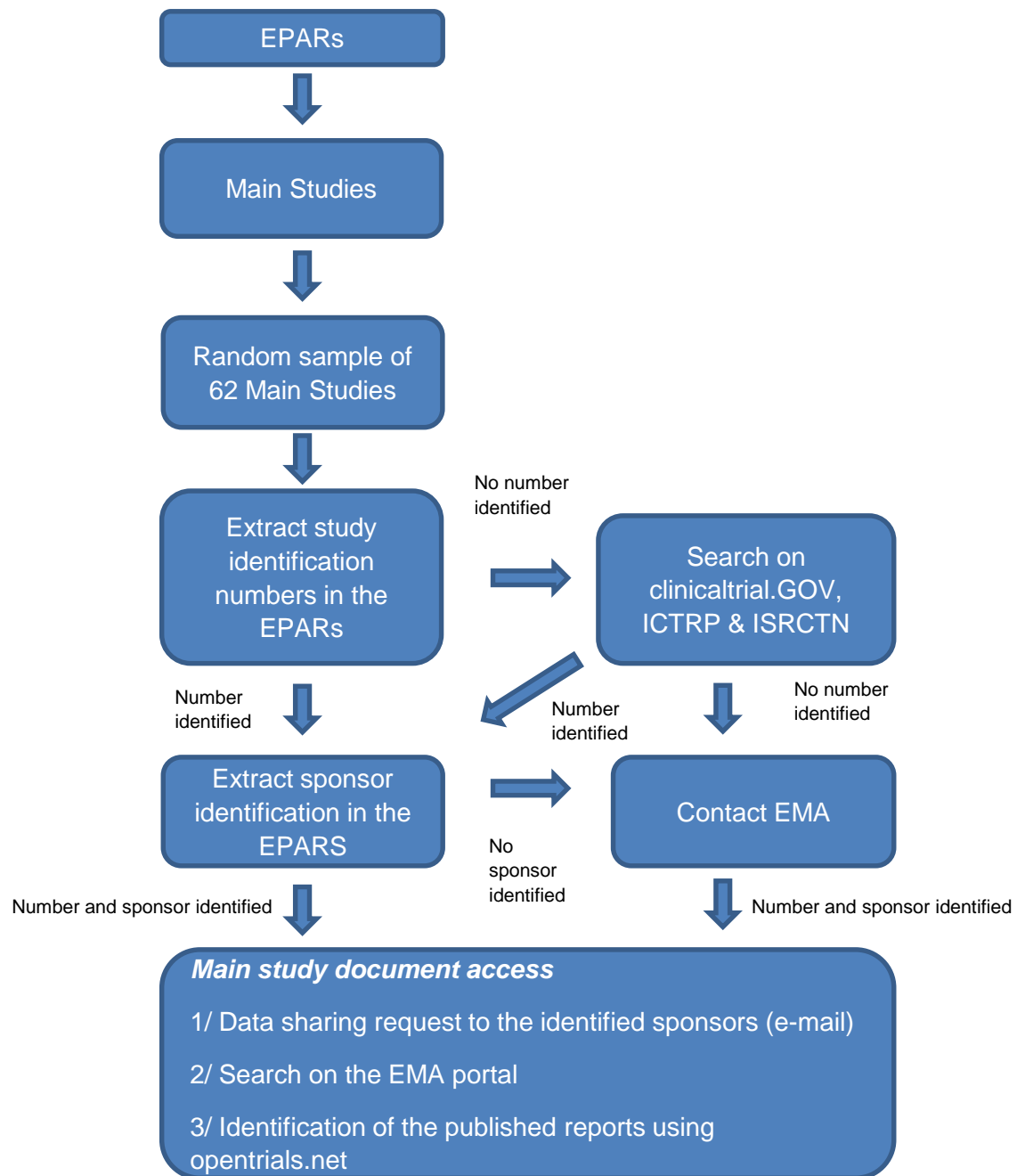

Supplement: Supplementary file 3 — Additional file 3: Figure S1 Process of accessing main study documents. [file 12916_2022_2377_MOESM3_ESM.pdf]

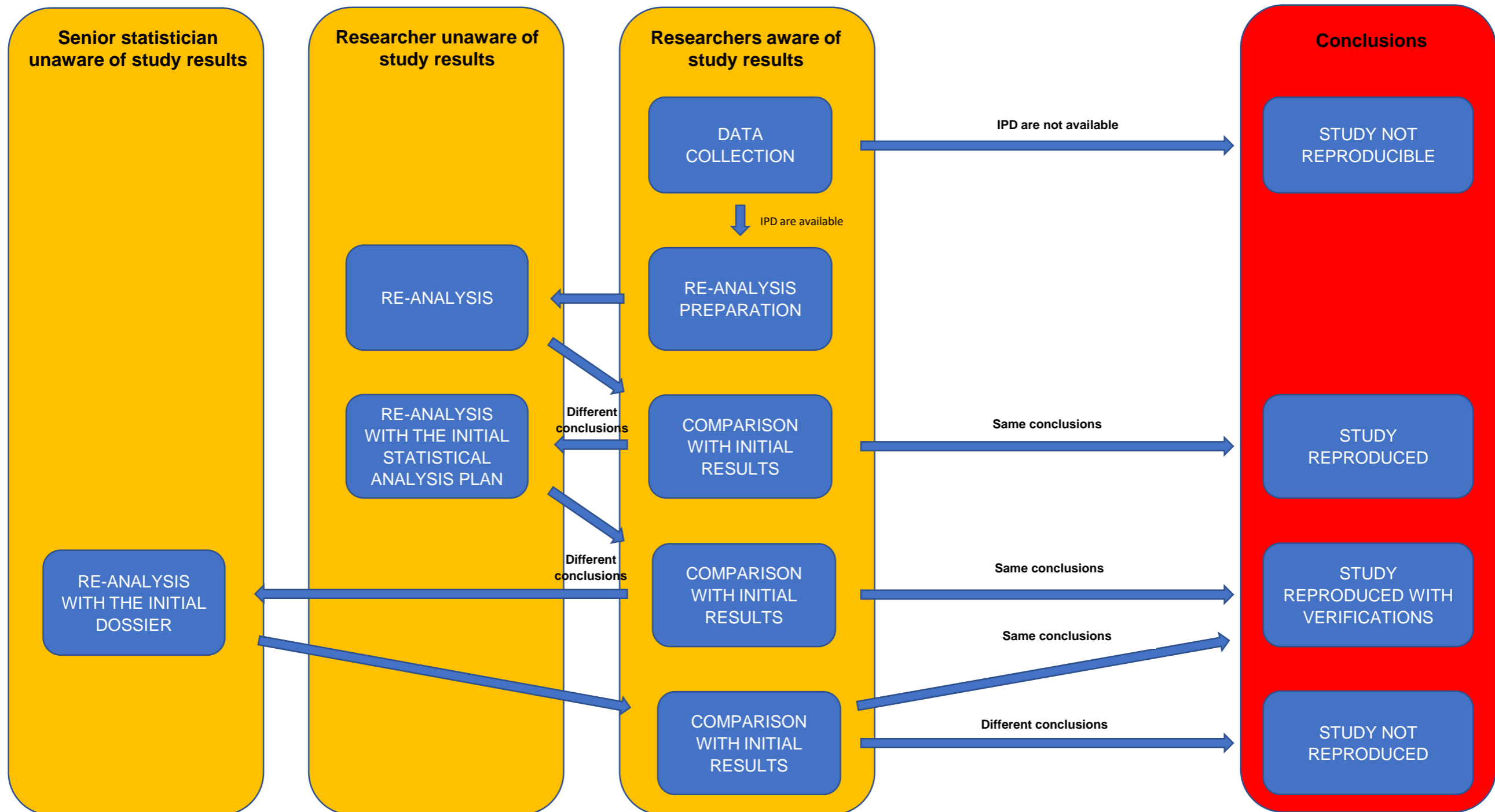

Supplement: Supplementary file 6 — Additional file 6: Figure S2 Procedure for assessing reproducibility. [file 12916_2022_2377_MOESM6_ESM.pdf]
