# Supplementary material for: Data-sharing and re-analysis for main studies assessed by the European Medicines Agency—a cross-sectional study on European Public Assessment Reports
Source: BMC Med. 2022 May 20;20:177. doi: 10.1186/s12916-022-02377-2 (PMC9119701; doi:10.1186/s12916-022-02377-2)
Supplement: Supplementary file 4 — Additional file 4: Table S2 Study Characteristics extracted. [file 12916_2022_2377_MOESM4_ESM.docx]

**Table of Study Characteristics that will be extracted**

| **Patient Characteristics** |  |
| --- | --- |
| Number of Participants | Quantitative information |
| Number of Groups | Quantitative information |
| Percentage of women | Quantitative information |
| Mean age of participants | Quantitative information |
| Pediatric indication | Binary information (Yes/No) |
| **Study Characteristics** |  |
| Year of publication (YYYY) | Quantitative information |
| Authors’ Name | Qualitative information |
| Country of study location (EU, North America, Asia, Other) | Qualitative information |
| PMID | Qualitative information |
| Trial Type (Cluster, Parallel, Cross-over) | Qualitative information |
| Trial Design (Superiority or non-inferiority or equivalence / head to head or another design (factorial)) | Qualitative information |
| Bias Assessment (according to Version 2 of the Cochrane risk-of-bias tool for randomized trials)   \| Selection Bias: Random Sequence Generation \| \| --- \| \| Selection Bias: Allocation concealment \| \| Performance Bias: Blinding of participants and staff \| \| Detection Bias: Blinding of outcome assessment \| \| Attrition Bias: Incomplete outcome data \| \| Reporting Bias: Selective reporting \| \| Other Bias Other: sources of bias \| | Qualitative information |
| Medical specialty | Qualitative information |
| Primary Endpoint Definition | Qualitative information |
| Primary Endpoint type (Clinical, surrogate or other) | Qualitative information |
| Additional Endpoints (in case of outcome switching) Definition | Qualitative information |
| Additional Endpoint (in case of outcome switching) types (Clinical, surrogate or other) | Qualitative information |
| Study Duration (Years, Standard Deviation) | Quantitative information |
| **Intervention Characteristics** |  |
| Intervention Drug | Qualitative information |
| Comparator drug (Placebo or another drug) | Qualitative information |
| Duration of exposure (Years, Standard Deviation) | Quantitative information |
| Industrial sponsorship  No  Device provided  Intervention provided  Drug provided  Drug and some financial support provided  Partial financial support provided  Total financial support provided | Qualitative information |
