# Supplementary material for: Data-sharing and re-analysis for main studies assessed by the European Medicines Agency—a cross-sectional study on European Public Assessment Reports
Source: BMC Med. 2022 May 20;20:177. doi: 10.1186/s12916-022-02377-2 (PMC9119701; doi:10.1186/s12916-022-02377-2)
Supplement: Supplementary file 5 — Additional file 5: Table S3 Details of information extracted from the ICH guidelines in case of missing information. [file 12916_2022_2377_MOESM5_ESM.docx]

**Table with details of what will be taken from the ICH guidelines in case of missing information**

|  |  | Information in the study documents | If not present, replace according to ICH section | Information used in the reanalysis |
| --- | --- | --- | --- | --- |
| Trial design | Design configuration |  | ICH E9, Point 3.1 |  |
|  | Type of comparison |  | ICH E9, Point 3.3  ICH E10 |  |
|  | Multicenter trial |  | ICH E9, Point 3.2 |  |
| Statistical analysis plan | Objectives and hypotheses |  | ICH E6 |  |
|  | Primary outcomes |  | ICH E9 |  |
|  | Secondary outcomes |  | ICH E9 |  |
|  | Sample size |  | ICH E9, point 3,4 |  |
|  | Methods to minimize bias |  | ICH E9, point 2.3  ICH E10, point 1.2 |  |
|  | Effect size |  | ICH E9, point 3.5 |  |
|  | Confidence interval |  | ICH E9, point 5.5 |  |
|  | Interim analysis |  | ICH E9, point 4.5 |  |
|  | Secondary analyses |  | - |  |
|  | Analysis method |  | ICH E9, point 5 |  |
|  | Handling missing data |  | ICH E9, point 5.3 |  |
|  | Hypothesis testing |  | ICH E9, point 5.5 |  |
|  | Statistical packages used for analysis |  | - |  |
|  | Protocol violations |  | ICH E9, point 2.3  ICH E6, point 6 |  |
|  | Analysis sets |  | ICH E9, point 5.2 |  |
